# Psychosocial impact of being diagnosed with mild cognitive impairment: patient and carer perspective

**DOI:** 10.1192/bjo.2023.499

**Published:** 2023-07-25

**Authors:** Nida Munawar, Liam Kennedy, Memoona Usman, Diana Burgui, Irene Bruce, David Robinson, Elaine Greene

**Affiliations:** Memory Clinic, Mercer's Institute for Successful Aging, St James's Hospital, Dublin, Ireland; and Trinity College Dublin, College Green, Dublin, Ireland; Memory Clinic, Mercer's Institute for Successful Aging, St James's Hospital, Dublin, Ireland

**Keywords:** Cognitive neuroscience, dementia, psychosocial interventions, social functioning

## Abstract

**Background:**

Mild cognitive impairment (MCI) may represent an intermediate, prodromal phase of dementia. Although persons with MCI (PwMCI) are able to function independently, they often experience reduced ability to carry out their usual activities. This can result in social, emotional and functional challenges.

**Aims:**

To explore the understanding and psychosocial impact of receiving a diagnosis of MCI on patients and carers.

**Method:**

A cross-sectional cohort study was conducted at St James's Hospital Memory Clinic involving patients who attended the clinic for assessment from 1 January 2020 to 30 April 2021 and received a diagnosis of MCI. We completed questionnaires with patients and a nominated family member or friend of each patient (FwMCI).

**Results:**

Forty-seven PwMCI participated in the study, and 36 nominated family members and/or friends completed the FwMCI questionnaire. In our cohort of PwMCI, most of the participants were not aware of their diagnosis; only 21% used the term MCI, and only 25% attributed their problems to a pathological cause. The majority of participants had no recollection of any discussion around the likelihood of progression. One-third of participants expressed relief that they did not have dementia. Most PwMCI reported positive psychological well-being and did not endorse symptoms of depression or anxiety. There was slight discordance of illness perception among the PwMCI–FwMCI dyads. Forty-seven per cent of FwMCI reported at least a mild degree of carer burden on the Zarit Burden Scale.

**Conclusions:**

Patients’ awareness of being diagnosed with MCI is relatively limited. Public education campaigns raising awareness about MCI can help influence the ‘illness representation’ for MCI and enable people to seek timely advice and support.

According to the World Health Organization in 2020, around 50 million people have dementia worldwide. This number is projected to reach 82 million in 2030 and 152 million in 2050. Mild cognitive impairment (MCI) is considered to be an intermediate, prodromal phase of dementia. Its prevalence will increase with the predicted ‘dementia avalanche’ due to increasing life expectancy.^1^

MCI is a heterogenous syndrome characterised by deficits in cognitive functioning (i.e. learning and memory, language, executive functioning, attention and social cognition) without significant impairment in instrumental activities of daily living.^2^ Although persons with MCI (PwMCI) are able to function independently, they often require some assistance with complex activities. This can result in social, emotional and functional challenges and consequent caregiver burden.^3^ The issue of caregiver burden has been extensively explored in dementia; however, caregiver burden may also be a significant issue in MCI, and the PwMCI–carer dyad should be supported for better outcomes.^4–6^ The recent Manchester consensus group for MCI recommends exploring the psychosocial impact of receiving a diagnosis of MCI on patients and carers.^2^

A substantial proportion of individuals seen in memory clinics are diagnosed with MCI; however, we have little objective data on the psychosocial impact of the diagnosis for these patients. In order to address this knowledge gap we designed a cross-sectional, cohort study. The study had the following aims.

- To examine the perspectives of PwMCI and family members and/or friends on their experience of receiving the results of an assessment at the memory clinic, including their recall and understanding of the diagnosis.
- To explore emotional well-being and changes in attitude to health and lifestyle following the diagnosis.
- To explore and understand the needs of the PwMCI and their family members.

## Method

### Service description

St James's Hospital (SJH) Memory Clinic provides multidisciplinary diagnostic assessments of complex cognitive presentations. The service receives referrals from multiple sources, both local and national, for primary and secondary opinions. Assessment at SJH Memory Clinic entails a comprehensive interview, collateral history and physical examination by a medical doctor, carried out in conjunction with neuropsychological testing. Diagnostic investigations including blood testing and neuroimaging (typically magnetic resonance imaging) are completed for all patients. Cases are discussed at a multidisciplinary consensus meeting, where expert opinions from neurology, geriatrics, psychiatry and neuropsychology are discussed to arrive at a diagnosis. Where diagnosis remains unclear or debatable, more in-depth investigation (such as cerebrospinal fluid biomarkers, functional neuroimaging or further targeted neuropsychological testing) is completed.

SJH Memory Clinic uses the MCI diagnostic label cautiously. The clinicians strive to further subtype MCI as amnestic or non-amnestic, single domain or multiple domain, with a comment on risk of progression.

The results of the assessment and associated recommendations are discussed with the patient at a face-to-face or telephone feedback meeting, depending on the patient's preferences and the complexity of the case. Patients are advised, if possible, to bring a family member to the meeting, and personalised written results and recommendations are also provided at the meeting for future reference.

At the feedback meeting, patients are advised about addressing potentially modifiable risk factors that can prevent or slow cognitive decline. The discussion focusses on well-described brain health measures. This includes evaluating lifestyle profile (activity, diet, smoking, alcohol use), social circumstances, vascular risk factors (hypertension, diabetes), sensory function (hearing, vision) and mental health (depression). Social prescribing networks and wellness programmes are signposted. Patients are also given written information about brain health measures.

### Study methodology

Ethical approval for the study was granted by SJH and Tallaght University Hospital Research Ethics Committee (project ID: 0344). The study was completed with patients who had attended the Memory Clinic for an assessment from 1 January 2020 to 30 April 2021 and had received a diagnosis of mild cognitive impairment, together with their nominated family member or friend. A time period of more than 12 months was used to account for the times when the Memory Clinic was not fully operational owing to COVID-19 restrictions. A list of the names of patients who had received a diagnosis of MCI (the PwMCI) was drawn from the clinic database. A patient information leaflet explaining the purpose and details of the study, along with a consent form, was posted to patients who had given consent to be contacted in the future for participation in research by the Memory Clinic team. A week later, the PwMCI were contacted by a member of the team to answer any questions about the study, obtain verbal consent and set a suitable date and time for a 15-min phone call to complete the questionnaire. Also, with patients’ consent, details of the family member or friend who was most involved in their lives and most familiar with their clinic evaluation were obtained, so that this individual could be invited to complete the carer questionnaire. The patients were requested to complete and sign the consent form and post it to the memory clinic using the stamped and addressed envelope sent to them. For the purpose of consistency, family members and friends who support PwMCI are referred to here as ‘carers’, although they may not have regarded themselves as such. We also use ‘FwMCI’ to refer to family members or friends of PwMCI. Information leaflets explaining the study and inviting FwMCI to participate were posted to the given addresses along with the consent forms. The same steps as described above were followed for FwMCI to complete the questionnaire over the phone. If a patient did not give consent to contact a family member or friend, or the family member or friend declined to participate in the study, then only the PwMCI's responses were included in the data-set for that case.

A questionnaire was formulated (using Microsoft Word, version 16, Windows) by iterative design, and the following variables were explored and recorded:

- Demographic details including age, gender, marital status, level of education and employment.
- Recall and understanding of the diagnosis and advice given following assessment at the memory clinic.
- Changes in life following the diagnosis, including lifestyle changes following doctor's recommendations.
- Psychological impact of the diagnosis, determined by enquiring about mood, self-esteem, and continued participation in enjoyable activities and by completing the Geriatric Anxiety Inventory – Short Form (sensitivity 75%, specificity 87% for anxiety disorders).^7^
- Identification of unmet needs e.g. more information about the diagnosis, support to implement advised lifestyle changes and future planning.

A similar questionnaire was formulated for FwMCI to record demographic details, recall and understanding of the diagnosis, degree of involvement with PwMCI and Zarit Burden Scale to assess carer burden.^8^ FwMCI were requested to complete and return the Zarit Burden Interview along with the consent form, and the rest of the questionnaire was completed over the phone.

This study used mixed methods, involving quantitative and qualitative data. The interviewer selected the most accurate option based on the person's response for each of the questions; if none of the options applied then the person's response was written down verbatim next to the ‘other’ option. Data were initially collected on study datasheets during the phone call, coded and then transferred to a Microsoft Excel (version 16, Windows) spreadsheet for analysis.

The authors assert that all procedures contributing to this work comply with the ethical standards of the relevant national and institutional committees on human experimentation and with the Helsinki Declaration of 1975, as revised in 2008.

## Results

**Fig. 1:**
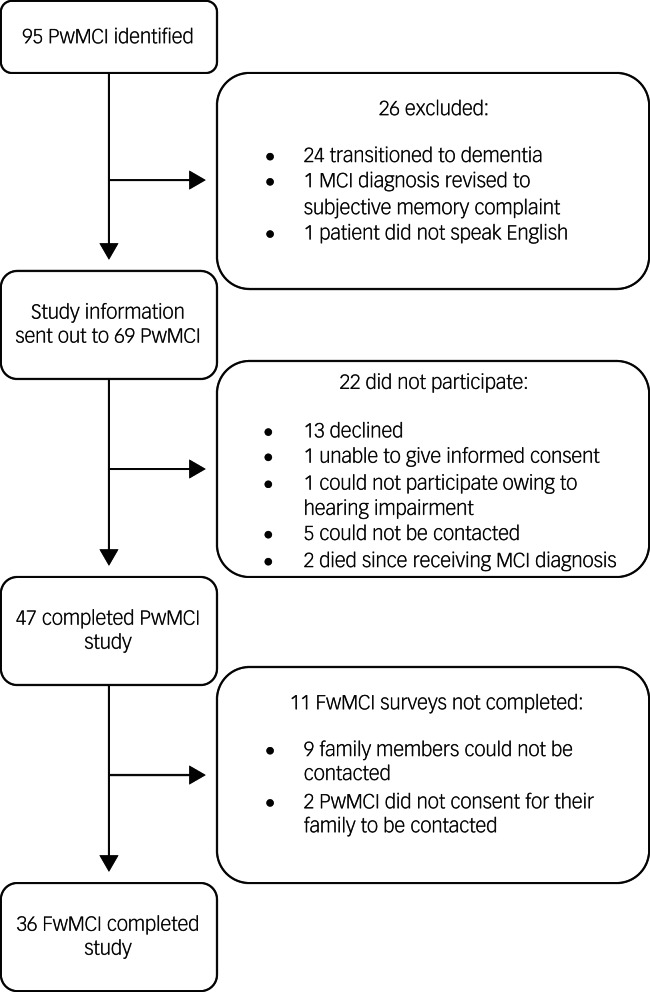
Flowchart detailing participant recruitment. MCI, mild cognitive impairment; PwMCI, person with MCI; FwMCI, friend or family member of person with MCI.

A total of 250 new patient assessments were carried out in the Memory Clinic from 1 January 2020 to 30 April 2021. Ninety-five patients (38%) received a diagnosis of MCI. Figure 1 details the recruitment of the participants for the study. Forty-seven PwMCI participated in the study (response rate: 68.1%).

### Patient characteristics

The demographic characteristics of the PwMCI cohort are presented in Table 1. The mean age of the participants was 71.6 years, and the median age was 71.0 years (range: 54 to 92 years). Most of the participants were retired (66%, *n* = 31). Only 8.5% of PwMCI (*n* = 4) reported the diagnosis affecting their working status. Of these, two took early retirement, one changed post and one reduced their working hours.

**Table 1:** Demographics of patients with mild cognitive impairment

Demographic characteristics	Percentage (*N*)
Gender	
Female	46.8 (22)
Male	53.2 (25)
Highest level of education attained	
Primary	21.3 (10)
Secondary	48.8 (23)
Third	23.4 (11)
Masters, PhD or similar	6.4 (3)
Marital status	
Married or civil partnership	57.4 (27)
Cohabiting	0
Widowed	23.4 (11)
Separated/divorced	8.5 (4)
Never married	10.6 (5)
Living status	
Alone	34 (16)
Spouse only	40.4 (19)
Partner	2.1 (1)
Spouse and children	14.9 (7)
Sibling	2.1 (1)
Religious community	2.1 (1)
Working status	
Full time	6.4 (3)
Part time	14.9 (7)
Not working	78.7 (37)
Health cover	
Medical card only	42.6 (20)
GP card only	12.8 (6)
Private health insurance only	27.6 (13)
Medical card and private health insurance	4.3 (2)
GP card and private health insurance	6.4 (3)
Passionist health insurance	2.1 (1)
No health cover	4.3 (2)

Most participants (59.6%, *n* = 28) had no personal experience of dementia through a diagnosis of a family member or friend. In most cases, the referral to the Memory Clinic was prompted by another person, i.e. a concerned family member (34%, *n* = 16), concerned general practitioner (GP) (25.5%, *n* = 12), another specialist (12.8%, *n* = 6) or a friend (*n* = 1), or following an in-patient stay (*n* = 1). In about one-fifth of the cases (*n* = 10) was the referral initiated owing to the PwMCI's own concerns. No correlation was found between observation of a relative or friend with memory problems and seeking a self-referral (*r* = 0.13).

On enquiry, most of the PwMCI were satisfied with their current health state and perceived it to be in the good-to-excellent range (excellent *n* = 5, very good *n* = 12, good *n* = 14), followed by 27.7% (*n* = 13) of PwMCI who reported it as fair. Only 6.4% (*n* = 3) perceived their current health to be poor. Most PwMCI (66%, *n* = 31) did not report a history of mental illness, and others were currently receiving treatment from a psychiatrist or community mental health team (19.2%, *n* = 9) or their GP (14.9%, *n* = 7).

### Experience of the memory clinic feedback appointment

On enquiry about the outcomes of the assessment, most participants (59.6%, *n* = 28) did not recall the diagnosis of MCI or a medical construct to explain their presentation. Many PwMCI (34.0%, *n* = 16) did not remember the details of the results discussed and did not view their problems as arising from a disease process. Only 21.3% (*n* = 10) of PwMCI used the term ‘mild cognitive impairment’ to describe their cognitive difficulties; 14.9% (*n* = 7) described these as ‘mild memory problems’, one participant said ‘mild impairment’ and one said ‘early dementia’. Most participants (57.4%) remembered discussing their results at a face-to-face meeting either with the doctor (*n* = 20) or a doctor and social worker (*n* = 7). Others said that the results were discussed by a doctor over the phone (*n* = 7, 14.9%), two participants said that they only received written summary of their assessment in the post and eight participants did not remember the format of feedback. There was no correlation found between face-to-face meeting and accurate recall of the diagnosis as ‘mild cognitive impairment’ (*r* = 0.3).

Only a quarter of the PwMCI (*n* = 12) viewed their problems as pathological and attributed them to a likely cause or narrative. The causes described included ‘brain haemorrhages’, ‘ECT related’, ‘amyloid in the brain’, ‘few different things’, ‘anxiety’, and ‘tablets prescribed for depression’. Many considered it to be due to ageing. More than half of PwMCI (60%, *n* = 28) said that likely causes were not discussed, and three participants said that they ‘don't remember’.

The participants had a varied understanding of the likelihood of progression of their difficulties. Most said that the chances of progression were not discussed with them (32%, *n* = 15) or that they did not remember whether it was discussed (29.8%, *n* = 14). About one-fifth of the PwMCI believed that their problems were unlikely to progress, and only four respondents understood that progression is determined by many factors. A similar number (*n* = 4) believed that their difficulties were highly likely to progress to dementia.

‘Relief that it's not dementia’ was the most common reaction of PwMCI to the results (34.0%, *n* = 16). Others said that they were glad to know the likely cause of their difficulties (14.9%, *n* = 7), six participants (12.8%) were shocked that their problems were more serious than they had anticipated and one participant reported being stigmatised with a label.

Most PwMCI (*n* = 30, 63.8%) were satisfied with the process of disclosure and with the advice given to manage their problems. The majority of the participants discussed the results with family only (53.2%, *n* = 25), followed by both family and friends (36.2%, *n* = 17). Some participants did not share the results of their assessment with friends or family (8.5%, *n* = 4). One participant said that this was owing to feeling embarrassed, and others said that there was ‘no diagnosis to share’.

When asked for suggestions for improving the diagnostic and disclosure process, only 17.0% (*n* = 8) of PwMCI had something to say. Some suggestions were as follows.

*‘Be definite and not just say it might not be that’*

*‘Would have preferred to receive the written personalised feedback form before the face-to-face meeting. It would have given me time to process the information and prepare questions. Final report that is sent to the GP should also be sent to the patient’*

*‘The team should keep in contact more frequently rather than just a yearly review’*

### Lifestyle changes after the diagnosis

**Fig. 2:**
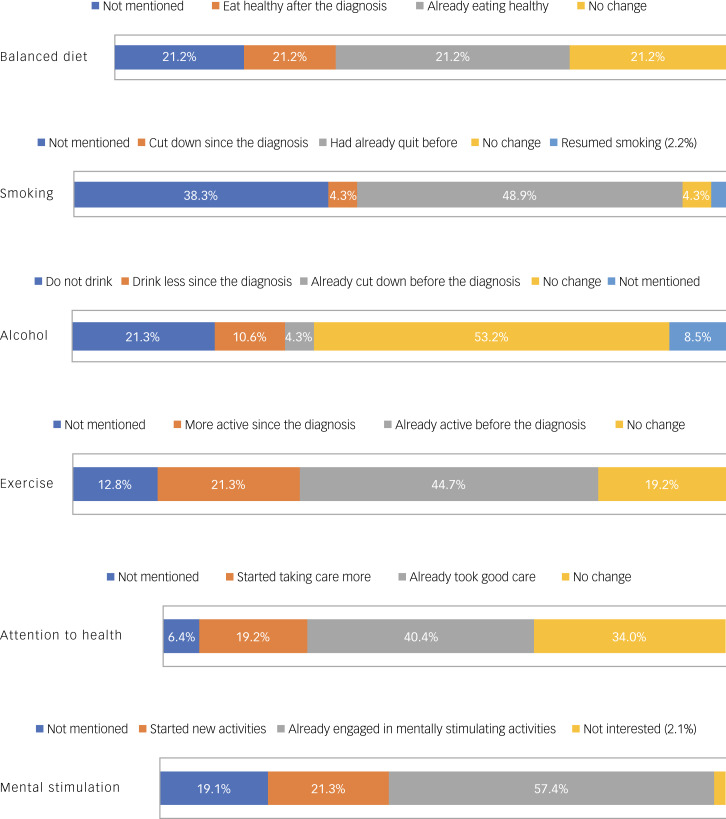
Lifestyle changes after diagnosis.

Figure 2 summarises the responses of the participants when asked about various aspects of their lifestyle following their attendance at the Memory Clinic and receiving brain health advice. ‘Doing crosswords’ and ‘reading’ were the most common cited mentally stimulating activities by PwMCI. When asked about new activities since the diagnosis, PwMCI had taken up golf (*n* = 1), playing football with kids (*n* = 1), writing (*n* = 1), doing arts (*n* = 1), crosswords and games (*n* = 1), reading and mindfulness (*n* = 2), resuming language classes (*n* = 1) and socialising with friends (*n* = 1).

Most of the participants (57.4%, *n* = 27) said that they had continued to drive with no problems since the diagnosis, three said that they are more cautious now and only one had quit driving since the diagnosis.

Awareness of hearing and vision as potentially modifiable risk factors was also explored. Most (55.3%) either didn't remember (*n* = 12) having a hearing screen during the assessment visit or said that it was not undertaken (*n* = 14). Among the others, 20 (42.6%) participants recalled having a hearing screen, of which six said that they were advised to have further audiology assessment. Only two PwMCI said that they were prescribed hearing aids following the audiology assessment, and only one participant reported wearing them all the time. Very few participants (17.0%, *n* = 8) recalled being informed that uncorrected visual problems can adversely affect the memory, and only one of them scheduled an optician review to mitigate the risk.

### Future planning

Many participants (61.7%, *n* = 29) had already written their Wills before the diagnosis. Only 10.6% (*n* = 5) proceeded to write their Will after the diagnosis. No correlation was seen between PwMCI perceiving that their condition can worsen in the future and writing a Will (*r* = 0.12). The attitude towards enduring power of attorney (EPOA) was varied. Many did not understand its significance (*n* = 16, 34.0%), others said that they had not set it up yet (*n* = 12, 25.5%) and only five PwMCI set up an EPOA after their diagnosis. Most participants (*n* = 44, 93.6%) did not make any changes to their living arrangements, and two participants said that they had been spending more time with their family since the diagnosis.

### Psychological impact

Most PwMCI (*n* = 28, 59.6%) responded by saying that the diagnosis had not affected their mood or self-esteem, and eleven participants (23.4%) acknowledged an effect to some extent. Most participants shared the views that the diagnosis probably had not changed the way other people see them (*n* = 31, 70%), that it was not affecting people close to them (*n* = 33, 70.2%), that they could control the progress of their problems (*n* = 34, 72.3%) and that they had not stopped doing anything they enjoyed since the diagnosis (*n* = 42, 89.4%). About one-fifth of PwMCI (*n* = 10) scored ≥3 on the Geriatric Anxiety Inventory – Short Form (sensitivity 75%, specificity 87% for anxiety disorders).^7^

### Family members study

The family member/friend part of the study could only be completed in 36 cases (Fig. 1). The mean age of FwMCI was 60.6 years and the median was 61.5 years. Seventy-eight per cent of the respondents (*n* = 28) were female, and the relationship of the respondents with PwMCI included spouse or partner (*n* = 18), child (*n* = 11), sibling (*n* = 6) and neighbour (*n* = 1). Twenty FwMCI (55.6%) were living with the PwMCI. Fourteen were working full time and five part time, and twelve were retired. Four FwMCI reported a change in their employment status since the diagnosis, and it was related to the diagnosis in three cases. Fifty-eight per cent of the respondents (*n* = 21) said that they also had other relatives or friends with memory problems or dementia.

#### Experience of the memory clinic feedback visit

Thirteen respondents (36.1%) attended a face-to-face feedback meeting at the Memory Clinic with PwMCI; seven (19.4%) were informed of the outcome of the assessment by the PwMCI themselves, and 12 (33.3%) were informed by the doctor over the phone, of whom four also read the written summary of the results sent out to the PwMCI in post. About half the FwMCI recalled the diagnosis as ‘mild cognitive impairment’ (*n* = 15); others recalled it as ‘minor cognitive impairment’ (*n* = 1), ‘cognitive impairment’ (*n* = 1) or ‘cognitive dysfunction’.^1^ About a quarter (*n* = 9) of the FwMCI said that they did not really know the diagnosis. Most participants had no insight into the likely cause(s) of PwMCI presentation: ‘wasn't told’ (*n* = 14), ‘the doctors do not know yet’ (*n* = 7), ‘cannot recall’ (*n* = 1). Fifteen (41.6%) FwMCI said that they were not informed about the chances of progression, and only a quarter of the respondents displayed an understanding of the possibility of the cognitive issues progressing.

About a third (*n* = 13) of FwMCI said that they were relieved that it was not dementia, whereas others expressed feeling reassured, surprised or shocked on hearing the results. Most FwMCI (*n* = 22, 61.1%) were satisfied with the information and advice given at the Memory Clinic. There were some suggestions for improvement that included providing more explanation and support to the family, clarity around role of medications and follow-up and signposting local supports and services suitable for the PwMCI at this stage.

#### Change in responsibilities

Sixteen FwMCI took up some responsibilities of the PwMCI around the time of the diagnosis. This most commonly involved financial planning (*n* = 8); other responsibilities included cooking, driving, medication management and looking after appointments. About the same number mentioned that they started helping the PwMCI more than 2 years ago. Only three PwMCI were receiving any outside help, e.g. carer or family visits. About two-thirds of FwMCI said that they had not discussed future planning or wishes with PwMCI yet. One quarter (*n* = 9) of FwMCI had additional care responsibilities in the form of caring for other family members or children. Most participants (*n* = 32) identified potential support, if needed in the future, involving other children, siblings, neighbours or extended family members of PwMCI.

#### Zarit Burden Interview

Thirty-four FwMCI completed the Zarit Burden Scale. Of those who scored in the burden range, 11 FwMCI reported mild to moderate burden, four reported moderate to severe burden and one reported severe burden.

## Discussion

People develop their own narratives to make sense of health states; these are known as ‘illness representations’. Illness representations determine the emotional response to a condition, as well as attitudes and behaviour regarding medical advice and treatment. Understanding these illness representations can guide clinicians about ways to best support their patients and provide personalised care.^9,10^

The Common Sense Model (CSM) developed by Leventhal et al^11^ proposes five dimensions that influence illness representations: (a) identity, (b) causes, (c) timeline, (d) consequences and (e) control. In our cohort of PwMCI, most of the participants did not recall the diagnosis of MCI for their condition. In addition, very few considered possible causes and fewer still considered that their condition might worsen in the future. Most participants described being in control of the progress of their condition and were relieved that it was ‘not dementia’. Overall, most patients did not report significant psychosocial distress or worry in relation to their MCI diagnosis. It may be argued that MCI is a more abstract illness concept than a diagnosis of dementia; this, in combination with the inherent memory difficulties in PwMCI, may account for the lack of strong MCI illness representation seen in our cohort.

There was slight discordance of illness perception among the PwMCI–FwMCI dyads. About half of FwMCI recalled a diagnosis to explain their loved one's symptoms, and about a quarter of FwMCI displayed reasonable insight into the possibility that the condition could worsen over time. Most FwMCI had limited understanding of the chances of progression or cause(s) of their loved one's condition and were relieved that it was not dementia. The CSM proposes that development of beliefs about a condition or illness is a dynamic process that is influenced by a person's previous experience of illness and treatment, observation of others close to them, social comparison and information from the media. More specifically, evidence suggests that prior personal experience of dementia in family or friends and perceived health status of an individual influences the way they interpret and cope with the MCI diagnosis.^12^ In our cohort, in contrast to FwMCI, the majority of PwMCI had no previous experience of a person with memory problems. This may explain the discordance in the perception of MCI between the two groups.

We found that a significant minority (21%) of patients diagnosed with MCI had self-instigated referral for assessment: the majority attended based on concern raised by family, friends or healthcare professionals. This is in keeping with our finding that overall FwMCI displayed greater understanding of the diagnosis of MCI than PwMCI themselves.

Beliefs about illness causality affect health-related behaviours and emotional response to an illness.^13^ Most participants in both groups did not attribute a likely cause to the PwMCI's presentation, and many considered it to be due to ageing. This is consistent with findings of earlier studies.^14^ Participants in our study did not attribute MCI to modifiable lifestyle risk factors such as physical inactivity, smoking, obesity, social isolation or poor diet and had limited understanding of the potentially modifiable risk factors for dementia. This is notable, as detailed information leaflets describing MCI and discussing lifestyle interventions are provided at diagnostic feedback meetings. It is possible that the PwMCI may be experiencing ‘information overload’ at these meetings. It is also possible that the PwMCI do not recognise the interventions suggested in the advisory leaflet as relevant to them. Finally, the level of understanding of the diagnosis may be related to the type of cognitive deficits the PwMCI is experiencing. We need to consider more impactful ways of educating PwMCI about modifiable risks. Simply providing information leaflets clearly does not meet their needs.

Very few participants in both groups were concerned about the condition progressing to dementia. This may explain the limited uptake of future planning advice provided at the feedback discussion and detailed in the feedback information pack. According to the CSM framework, a perception that illness is controllable is associated with positive psychological well-being.^15^ Most PwMCI in our study reported being in control of their memory problems. The majority reported positive psychological well-being and did not endorse symptoms of depression or anxiety. Patients who view their cognitive decline as part of ageing and consider it to be non-pathological score higher on measures of well-being and satisfaction with life.^9^ This was true for our PwMCI cohort, as many had not pathologised their cognitive problems. This raises the question of whether a greater understanding of the diagnosis of MCI and possibility of progression to dementia may cause more iatrogenic psychological harm than benefit.

Existing evidence suggests that patients’ and carers’ understanding of MCI is influenced by clinicians’ understanding and communication of the construct and quality of information provided.^12^ Clinicians have varying perspectives about the utility of the ‘mild cognitive impairment’ diagnosis. This influences their discussion of the results of the assessment and the advice given.^16,17^ Clinicians from different specialties and with different levels of clinical experience review patients in the Memory Clinic at SJH. The format of feedback meetings also varies depending on both the clinician's judgement and the patient's preferences. For this study, no data about the clinician's specialty and years of experience were collected. Hence, it is not possible to comment on any correlation between the clinician communicating the results and the participant's perspective in this study.

Although this study provides useful insights into the subjective experiences of PwMCI–FwMCI dyads, the results should be interpreted in the light of the study's limitations. The study recruited participants from only one clinic sample, the MCI diagnosis was not further categorised to group participants (type, neuropsychological scores) and the duration of MCI diagnosis varied among the participants. Cognitive changes inherent to MCI can affect a person's understanding and interpretation of the information provided, and participants may forget the information they have been told over time.^18^ Moreover, the study did not account for variation in the communication practices of the clinicians. Although the median age of our study participants was 71 years, this should not have a bearing on the generalisability of the findings, as participants’ ages varied widely. It can be argued that studying an older cohort of PwMCI may result in further insights, owing to complex interactions between cognitive impairment and frailty in older people.

The results of this study indicate several possible improvements for future policy and practice. Clinicians need to be more specific about underlying causes, prognosis and risk reduction interventions when communicating a diagnosis of MCI. It should be clearly stated that the patients’ cognitive difficulties are not normal for their age and education level. Many participants in our study expressed frustration at the lack of available treatments and services suitable for PwMCI. All patients receiving the diagnosis of MCI should be offered post-diagnostic support personalised to their needs in the form of psychosocial interventions and caregiver support.

About one-third of FwMCI in our study reported mild to moderate burden caused by supporting their loved one with MCI. Public education campaigns raising awareness about MCI and modifiable risk factors for dementia can help influence ‘illness representation’ for MCI. Based on our findings, awareness campaigns targeted at family and friends identifying MCI in their loved ones may be more effective than those encouraging people to seek assessment for subjective memory concerns. Timely intervention at the MCI stage can delay the onset of dementia and even improve cognitive abilities, resulting in better quality of life for PwMCI. This will reduce the burden on carers and the healthcare system in caring for people with dementia and potentially result in significant cost savings.

## Data Availability

The data that support the findings of this study are available from the corresponding author, N.M., upon reasonable request.
